# MicroRNA-221 silencing attenuates the degenerated phenotype of intervertebral disc cells

**DOI:** 10.18632/aging.101525

**Published:** 2018-08-20

**Authors:** Letizia Penolazzi, Elisabetta Lambertini, Leticia Scussel Bergamin, Tosca Roncada, Pasquale De Bonis, Michele Cavallo, Roberta Piva

**Affiliations:** 1 Department of Biomedical and Specialty Surgical Sciences, University of Ferrara, Ferrara, Italy; 2 Department of Neurosurgery, S. Anna University Hospital, Ferrara, Italy; 3 Center for Studies on Gender Medicine, University of Ferrara, Ferrara, Italy

**Keywords:** intervertebral disc cells, intervertebral disc degeneration, gene silencing, microRNA-221, FOXO3

## Abstract

The aim of this study was to investigate the role of an antichondrogenic factor, MIR221 (miR-221), in intervertebral disc degeneration (IDD), and provide basic information for the development of a therapeutic strategy for the disc repair based on specific nucleic acid based drugs, such as miR-221 silencing. We established a relatively quick protocol to minimize artifacts from extended in vitro culture, without selecting the different types of cells from intervertebral disc (IVD) or completely disrupting extracellular matrix (ECM), but by using the whole cell population with a part of resident ECM. During the de-differentiation process miR-221 expression significantly increased. We demonstrated the effectiveness of miR-221 silencing in driving the cells towards chondrogenic lineage. AntagomiR-221 treated cells showed in fact a significant increase of expression of typical chondrogenic markers including COL2A1, ACAN and SOX9, whose loss is associated with IDD. Moreover, antagomiR-221 treatment restored FOXO3 expression and increased TRPS1 expression levels attenuating the severity grade of degeneration, and demonstrating in a context of tissue degeneration and inflammation not investigated before, that FOXO3 is target of miR-221. Data of present study are promising in the definition of new molecules useful as potential intradiscal injectable biological agents.

## Introduction

Defective homeostatic mechanisms and accumulation of molecular damages in spine injuries and spine disorders must be elucidated. A particularly complicated scenario is represented by intervertebral disc degeneration (IDD), a multifactorial disease without effective preventive and therapeutic approaches [1,2]. The complex cellular fibrocartilaginous structure and mechanical environment of the intervertebral disc (IVD) make it difficult to obtain unequivocal data and set up appropriate/informative experimental models [3]. Consequentely, many studies which are mainly aimed at developing novel therapeutics based on the local injection of cells or biological agents for IVD repair produce conflicting data.

The IVD is composed of a hydrophilic proteoglycan-rich gelatinous core, the nucleus pulposus (NP), which is surrounded by a lamellated collagenous ring, the annulus fibrosus (AF), and cartilaginous and bony end-plates that separate the disc from the vertebrae [3]. Degeneration begins when anabolic and catabolic activities of IVD mature and progenitor cells become unbalanced due to negative stimuli including genetic risk, mechanical trauma, injuries, smoking, obesity and ageing [4,5]. This causes a change in tissue architecture, cell density and extracellular matrix (ECM) composition; the nucleus infiltrates the annulus and the cellular components mix together. Consequently, a variety of cells coexist in the degenerated microenvironment such as neurons, chondrocytes, and osteoblasts which come from both surrounding spinal tissue or differentiation of progenitor cells resident in the disc [1,2,5]. Therefore, when investigating IDD local microenvironment it must take into account the difficulties of both acquiring a uniform IVD tissue or obtaining homogeneous cell sub-populations. However, in a scenario like this it is not always necessary/convenient to sort single cell populations, but rather to try to preserve in vitro the properties of the endogenous microenvironment to obtain informative results. Therefore, the idea of not selecting the different types of cells, but of using the whole cell population with a part of resident ECM, is becoming increasingly convincing. Following this hypothesis, we are interested in understanding the endogenous properties of IVD cells and investigating the effectiveness of nucleic acid based drug treatments in the reverting degenerated phenotype.

In recent years, an increasing number of reports have described microRNAs (miRNAs) as key players in IDD [6–9]. Some miRNAs have been associated with apoptosis, ECM degradation, cell proliferation and senescence, oxidative stress and inflammation that are well known in promoting and maintaining IDD. Therefore, in addition to diagnostic and prognostic markers, miRNAs have also been proposed as potential therapeutic targets in order to promote disc repair [5]. Previously, we showed that antimiR-mediated silencing of MIR221 (miR-221) in human mesenchymal stem cells (hMSCs) functions as a potent pro-chondrogenic signal both in vitro and in vivo, enhancing chondrogenic markers and formation of new cartilage [10,11]. Here we examined, for the first time, the effectiveness of antagomiR-221 treatment in reverting the degenerated/de-differentiated phenotype of cells from enzymatically-dispersed low passage-expanded human IVD cells. At the same time, this knockdown approach allowed us to investigate potential targets of miR-221 in a context of tissue degeneration and inflammation not investigated before, providing basic information needed for the development of effective therapies mainly based on intradiscal injection of biochemical agents.

## RESULTS

### Cells from IVD: culturing and characterization

**Figure 1:**
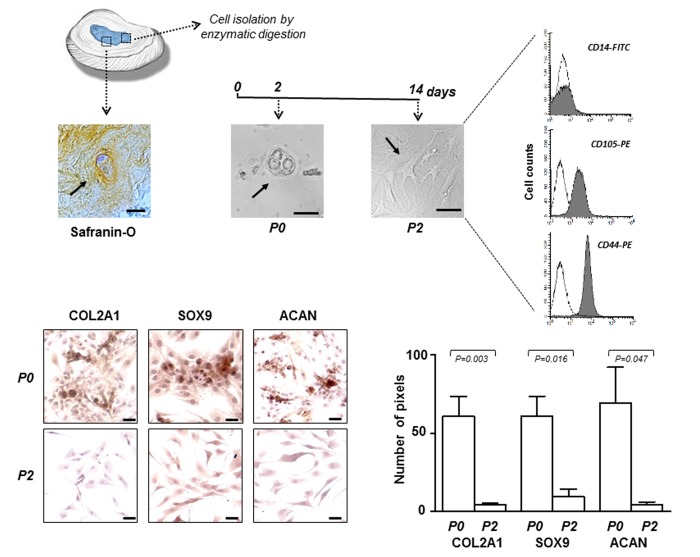
**IVD cells: culturing and characterization.** Representative optical photomicrographs showing morphology of the cells (indicated by the arrows) in the IVD tissue stained with Safranin-O, and at P0 and P2 passages in culture. P2 cells were characterized by flow cytometry for the expression of CD14 haematopoietic marker, and CD105 and CD44 mesenchymal markers. Flow cytometric analysis of a representative case is reported; open histograms represent the isotype control antibody, gray histograms represent anti-CD14, -CD105 and -CD44 antibodies. X-axis, fluorescent channel; Y-axis, number of events. Representative optical photomicrographs of COL2A1, SOX9 and ACAN immunostaining performed in P0 and P2 cells are reported. Protein expression levels were quantified by densitometric analysis of immunocytochemical pictures using ImageJ software and expressed as means of pixels per one hundred cells ±SD (P0 group, n = 6; P2 group, n = 6). Exact P-values are reported. Scale bars: 20 μm.

The experimental procedure to obtain IVD cells has been described in the Material and Methods section and in Table 1 the characteristics of the IDD patients have been reported. All tissue samples were assessed by histology (hematoxylin and eosin) and histochemistry (Safranin-O) revealing the presence of matrix proteoglycans in hypocellular areas, as shown in the representative microphotograph of Figure 1. Passage zero (P0) cells showed a morphology very similar to that found in the histological preparation and, as expected, changed in expanded P2 cells, where the predominant form became the flattened one (Figure 1).

**Table 1:** Human IVD specimen information.

	***IVD level***	***Age***	***Sex***	***Symptoms***	***Duration of symptoms prior to surgery***	***Degeneration***
***Donor 1***	C5-C6	40	female	Tetraparesis (myelopathy)	9 months	mild
***Donor 2***	C5-C6	47	male	Radiculopathy: pain and palsy	1 month	mild
***Donor 3***	C5-C6	63	female	Radiculopathy: pain and palsy	3 months	mild
***Donor 4***	C6-C7	48	male	Radiculopathy: pain and palsy	2 months	mild
***Donor 5***	C5-C6	64	female	Radiculopathy: pain and palsy; neck pain	3 months	severe
***Donor 6***	C5-C6	49	female	Radiculopathy; neck pain	2 months	severe
***Donor 7***	C4-C5	34	male	Paraparesis (myelopathy) and radiculopathy: pain and paresthesia; neck pain	6 months	severe
***Donor 8***	C6-C7	41	male	Radiculopahty: pain and paresthesia	3 months	severe
***Donor 9***	C5-C6	48	male	Paraparesis (myelopathy)	12 months	severe
***Donor 10***	C7-D1	34	male	Radiculopathy: pain and palsy	2 months	mild
***Donor 11***	C4-C5	68	male	Tetraparesis (myelopathy)	12 months	severe
***Donor 12***	C5-C6	40	female	Radiculopathy: pain	2 months	mild
***Donor 13***	C6-C7	55	male	Radiculopathy: pain and palsy	6 months	severe
***Donor 14***	C4-C5	70	male	Tetraparesis (myelopathy)	3 months	severe
***Donor 15***	C4-C5	58	male	Radiculopathy: pain and palsy	6 months	severe
***Donor 16***	C5-C6	44	male	Radiculopathy: pain and palsy	6 months	mild

After subculture, already at passage 2 (P2), the cells have undergone a de-differentiation process by loosing the chondrocyte-like phenotype. This was assessed by immunocytochemical analysis which revealed a sharp decrease in the expression of typical chondrogenic markers, including collagen type II alpha 1 chain (COL2A1), SRY-box 9 (SOX9) and aggrecan (ACAN) (Figure 1). Immunophenotypic profile of P2 cells from each culture was determined by flow cytometry showing that the cells were negative for CD14 haematopoietic cell marker, and positive for CD105 (80% ± 8%) and CD44 (89% ± 5%) mesenchymal cell markers (Figure 1). We chose to use P2 cells since they represent a good compromise as de-differentiated but no senescent cells, suitable for the subsequent efficient transfection.

### miR-221 expression in IVD cells

**Figure 2:**
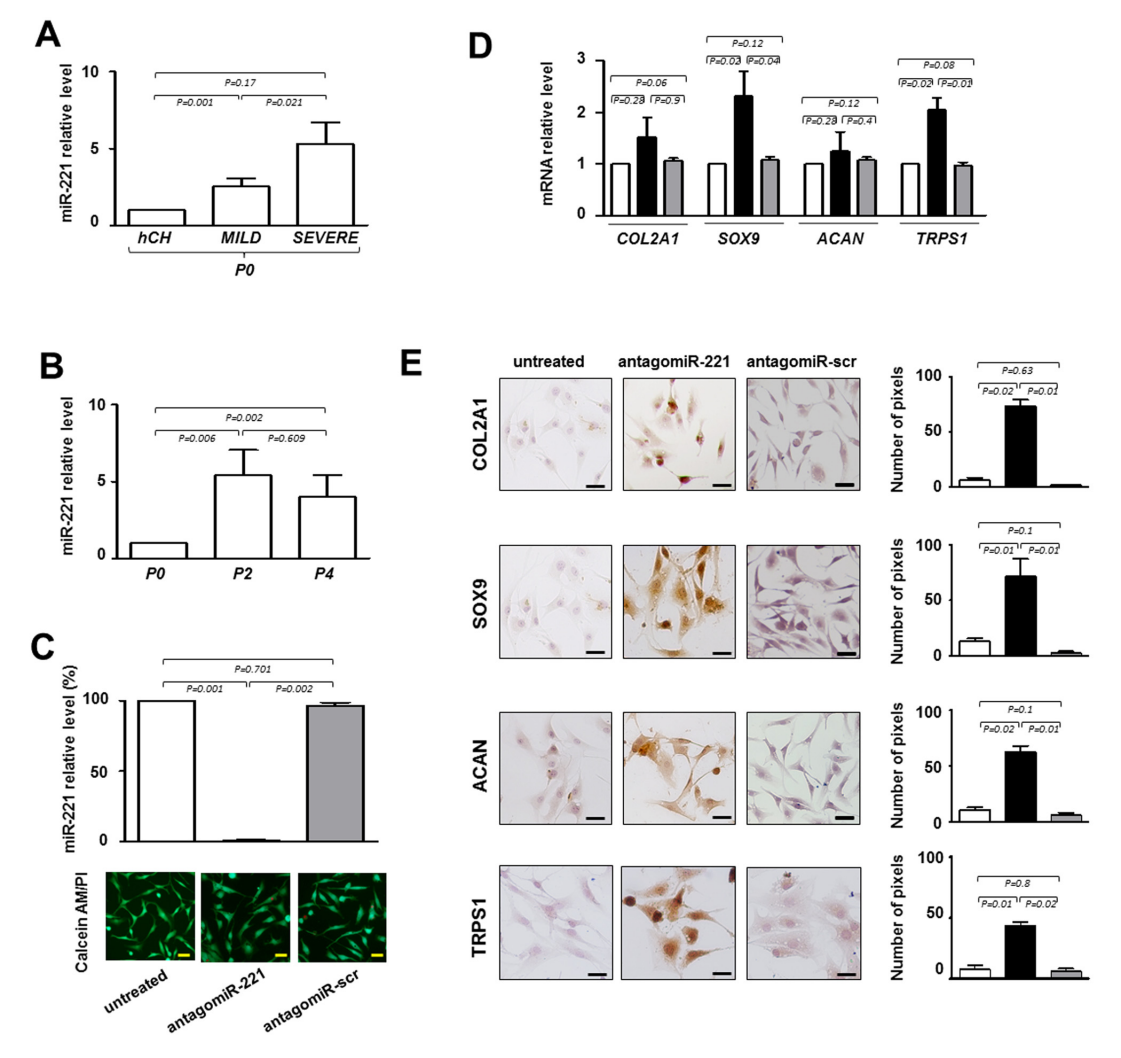
**Evaluation of the effect of antagomiR-221 treatment on intervertebral disc cells (IVD) cells.** (**A**) Before transfection the expression levels of miR-221 were measured in P0 cells from MILD and SEVERE IDD by qRT-PCR. Data are presented as fold changes relative to control represented by human freshly isolated chondrocytes (hCH). Results represent mean ± SD (hCH group, n = 6; MILD group, n = 7; SEVERE group, n = 9). Exact P-values are reported. (**B**) miR-221 levels were measured during de-differentiation process from passage 0 (P0) to passage (P4). Data are presented as fold changes relative to P0 cells. (P0 group, n = 6; P2 group, n = 6; P4 group, n = 6). Exact P-values are reported. Monolayered P2 cells were then transfected with antagomiR-221 (black column), a scrambled oligonucleotide (antagomiR-scr) (gray column) or remained untreated (white column) (**C**, **D**, **E**). (**C**) The efficiency of miR-221 knockdown was determined by qRT-PCR (**C**) and data are presented as fold change respect to control untreated cells ±SD (n = 10). Exact P-values are reported. Cell viability of transfected cells was determined by double staining with Calcein-AM/propidium iodide. The green fluorescence indicates the presence of calcein-labeled live cells, while propidium iodide-labeled dead cells are revealed by red fluorescence. Merged photomicrographs are reported. Scale bars: 20 μm. Expression of COL2A1, SOX9, ACAN and TRPS1 chondrogenic markers was assessed by qRT-PCR **D**) and immunocytochemistry **E**). (**D**) The expression of typical chondrogenic markers evaluated at mRNA level. mRNA data are presented as fold change relative to untreated cells. Results represent means ± SD (n = 10). Exact P-values are reported. (**E**) The expression of typical chondrogenic markers evaluated at protein level. Representative optical photomicrographs of COL2A1, SOX9, ACAN and TRPS1 immunostaining are reported. Protein levels were quantified by densitometric analysis of immunocytochemical pictures using ImageJ software and expressed as means of pixels per one hundred cells ±SD (n = 10). Exact P-values are reported. Scale bars: 20 μm.

Repeated analyses on IVD cells demonstrated that the expression of miR-221, previously defined as negative regulator of chondrogenesis [10] and mediator of inflammatory pathway [12], increased with the degree of degeneration. Interestingly, we found that the expression levels of miR-221 were always significantly higher in IVD cells than in normal human freshly isolated chondrocytes [10] used as control, and in P0 cells from severe IDD than P0 cells from mild IDD (Figure 2A). Consistently, the expression levels of miR-221 significantly increased during the de-differentiation process; P2 or P4 cells showed higher levels of miR-221 than P0 cells (Figure 2B).

These data suggest that degeneration and de-differentiation are two processes that, for certain aspects, can be overlapped acting through potential common regulators such as miR-221.

### miR-221 silencing in IVD cells

Next, we knocked down miR-221 by siRNA in de-differentiated P2 cells to determine whether miR-221 silencing was effective in inducing the expression of chondrogenic factors in the absence of exogenously added growth factors, as we previously observed in human mesenchymal stem cells (hMSCs) [10,11]. As shown in Figure 2C, antagomiR-221 treatment was highly effective in miR-221 knockdown after only 48 hours, achieving >99% inhibition of miR-221 expression with respect to untreated or antagomiR-scrambled treated cells. After transfection with antagomiR-221 or antagomiR-scrambled, cell viability was not modified as determined by Calcein‐AM/PI assay (Figure 2C).

Forty-eight hours of antagomiR-221 treatment was sufficient in modifing the expression of typical chondrogenic markers such as COL2A1, SOX9, and ACAN (Figure 2D and E). In particular, SOX9 was up-regulated both at mRNA and protein level. COL2A1 and ACAN mRNAs remain relatively unaffected, whereas protein expression significantly increased as revealed by qRT-PCR and immunocytochemistry analysis, respectively. The lack of correspondence between mRNA and protein levels for extracellular matrix components is not surprising [13,14] and this evidence confirms once again that protein analysis is preferable to obtain informative data in this context.

Interestingly, antagomiR-221 treatment was also effective in increasing the expression of TRPS1 (transcriptional repressor GATA binding 1), a repressor of calcification and a positive modulator of chondrocyte proliferation and differentiation, still poorly investigated in IDD (Figure 2D and E) [15].

Collectively, these data suggest that the silencing of miR-221 is critical for addressing the IVD cells towards a chondrogenic like phenotype.

### miR-221 silencing correlates with FOXO3 up-regulation

**Figure 3:**
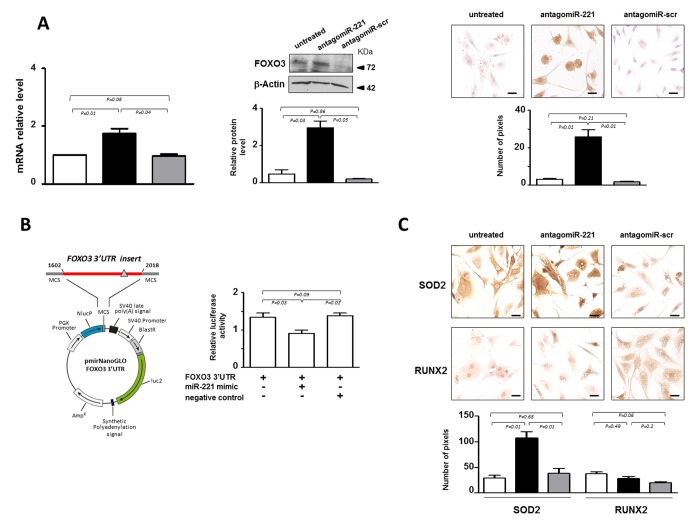
**miR-221 targets FOXO3 in IVD cells.** (**A**) Evaluation of the effect of antagomiR-221 treatment on FOXO3 expression. The expression levels of FOXO3 were assessed in antagomiR-221 treated de-differentiated P2 cells by qRT-PCR, Western blot and immunocytochemistry. mRNA data are presented as fold change relative to untreated cells. Results represent mean ± SD (n = 10). Exact P-values are reported. Representative Western blot of FOXO3 protein analysis is reported. Bar graphs show the densitometric analysis of all samples analyzed; β-Actin was used as loading control, data were expressed as ratio of FOXO3 in respect to β-Actin and presented as mean ± SD (n = 5). Representative optical photomicrographs of FOXO3 protein expression and localization assessed by immunocytochemistry are reported. Protein levels were quantified by densitometric analysis of immunocytochemical pictures using ImageJ software and expressed as means of pixels per one hundred cells ±SD (n = 10). Exact P-values are reported. Scale bars: 20 μm. In the graphs: antagomiR-221 (black column), scrambled oligonucleotide (antagomiR-scr) (gray column) treated or untreated (white column) cells are reported. (**B**) Validation of miR-221 target site in the FOXO3 3’-UTR by reporter gene assay in IVD cells. A luciferase reporter vector containing partial sequence (+1602/+2018) of the FOXO3 3’-UTR harboring the predicted miR-221 target site (gray triangle), in the 3’ UTR of a Nano Luc luciferase gene was used. P2 cells were transfected for 48 hours with a combination of reporter constructs (100 ng) along with miR-221 mimic or Negative control (30 nM). Afterwards, Nano Luc luciferase reporter gene (NlucP) and Firefly luciferase control reporter activities (luc2) were measured using a Nano-Glo Dual-Luciferase assay and represented as mean ±SD (n = 5). Exact P-values are reported. (**C**) The expression of SOD2 and RUNX2 was assessed by immunocytochemistry. Representative optical photomicrographs are reported. Protein levels were quantified by densitometric analysis of immunocytochemical pictures using ImageJ software and expressed as means of pixels per one hundred cells ±SD (n = 10). Exact P-values are reported. Scale bars: 20 μm. In the graphs, antagomiR-221 (black column), scrambled oligonucleotide (antagomiR-scr) (gray column) treated or untreated (white column) cells are reported.

In our effort to elucidate the molecular mechanisms supporting miR-221 action in IVD cells, we focused on the effect of antagomiR-221 treatment on FOXO3, a member of forkhead ‘O’ class transcription factors that have been recently defined as critical mediators of IVD integrity and function [16,17]. Computational analysis performed with three miRNA databases (miRanda, DIANA-microT v5.0 and miRTarBase) on the 3′ UTR sequence of FOXO3 identified possible binding sites for miR-221. It has already been shown, in a different cellular context, that miR-221 targets FOXO3 [18]. We found that in most cases analyzed de-differentiated P2 cells were completely FOXO3 negative. However, the expression of FOXO3 analyzed both at mRNA and protein level significantly increased in antagomiR-221 treated P2 cells (Figure 3A). The immunocytochemistry showed that the antagomiR-221 treated cells expressed functional FOXO3 transcription factor as it was found immunolocalized mostly in the nucleus.

The effect of miR-221 in regulating FOXO3, was further investigated by reporter gene assays (Figure 3B). 3’-UTR DNA fragment containing the validated miRNA target site of the gene [18] was generated by PCR and cloned into the 3’-UTR of a luciferase reporter gene. Forty-eight hours after cotransfection of P2 cells with reporter gene and miR-221 mimic, a significant down-regulation of the reporter gene activity was found (Figure 3B).

In order to investigate whether the increase of FOXO3 affected transcriptional programming, the expression of two target genes, manganese-containing superoxide dismutase (SOD2) [19] and runt-related transcription factor (RUNX2) [20], were evaluated. Previous studies indicated that FOXO3 is implicated in the detoxification of reactive oxygen species (ROS) through induction of SOD2, an enzyme with an important role in cellular stress responses [19]. Consistently, a remarkable increase of SOD2 expression was found following antagomiR-221 treatment (Figure 3C), showing that the increase of FOXO3 mediated by miR-221 silencing is accompanied by an important effector of FOXO signaling such as SOD2. In addition, we found that antagomiR-221 treatment maintained low basal expression levels of RUNX2 (Figure 3C). RUNX2, the master regulator of osteogenesis [20], was recently implicated in the progression of IVD aging, degeneration and calcification [21]. The range of RUNX2 action is wide and the regulation of its expression depends on many different factors, including FOXO3 and TRPS1, whose activity is strictly controlled by the cellular context. Therefore, the increase of SOD2, but not RUNX2, expression suggests the achievement of a downstream signaling that contributes to revert the de-differentiated phenotype of P2 cells after antagomiR-221 treatment.

### FOXO3 and TRPS1 expression in IVD specimens

**Figure 4:**
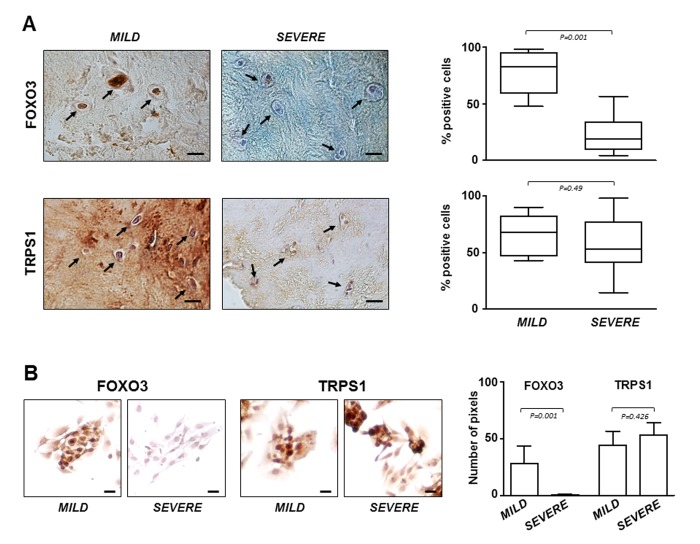
**FOXO3 and TRPS1 expression in IVD tissues and P0 cells.** (**A**) Immunohistochemistry on IVD tissues with MILD or SEVERE IDD and (**B**) immunocytochemistry on P0 cells demonstrating the presence of FOXO3 and TRPS1 (positive cells in representative optical photomicrographs are indicated with arrows). Protein levels were quantified by densitometric analysis of immunostaining using ImageJ software and expressed as % of positive cells per area (5 sections per sample; MILD group, n = 7; SEVERE group, n = 9). The results are reported as Whisker box plot representing min to max (line indicates median). For densitometric quantification of immunocytochemical pictures data were expressed as means of pixels per one hundred cells ±SD (MILD group, n = 7; SEVERE group, n = 9). Exact P-values are reported. Scale bars: 20 μm.

The critical role of FOXO3 in IDD was confirmed by immunostaining performed on all IVD tissue samples and P0 cells derived from them. The results showed that the lowest expression levels of FOXO3 were in severe degenerated discs (Figure 4A), as well as in the corresponding P0 cells compared to P0 cells from mild IDD (Figure 4B). Conversely, TRPS1 was expressed at comparable levels regardless of the degree of IDD, both in IVD tissue and in P0 cells (Figure 4A and B). This evidence supports the idea that P0 cells can be considered an adequate experimental model mimicking the IDD since resemble the characteristics of IVD from which derived. Moreover, restoring high levels of FOXO3 by miR-221 silencing can attenuate disease severity.

## DISCUSSION

Understanding molecular mechanisms involved in IDD is essential for developing treatments to prevent the onset and progression of IDD, in particular those treatments based on injectable bioactive molecules within the injured disc.

We found that miR-221 is up-regulated in cells from degenerated IVD and the aim of this study was to investigate the effect of antagomiR-221 treatment in reverting the degenerated phenotype of these cells.

In addition to classic gene therapies and cell-based tissue engineering approaches [5,22–25], in recent years several treatments have been proposed in preclinical setting predominantly consisting of direct injection of biological agents, ranging from recombinant growth factors and matrix proteins [26–30], to Platelet-rich plasma (PRP) [31], to nucleic acid based drugs including a variety of long and small non-coding RNAs (ncRNAs) [5,32]. Among housekeeping and regulatory ncRNAs, miRNAs are important small ncRNAs regulators of gene expression, and act by targeting mRNAs for translational repression and/or cleavage [33]. Increasing evidence shows that strategies to downregulate a specific miRNA, or to prevent its upregulation, have potential as therapeutic and/or preventive approaches in management of a variety of human diseases including IDD. However, conflicting data or unconvincing results are present in literature, mainly due to the difficulty of setting up appropriate in vitro or in vivo experimental models. A significant differential expression of some miRNAs between normal and degenerative NP tissues, and an association with the development and progression of IDD have been demonstrated, and many knockdown or overexpression experiments have been performed in vitro. For example, it has been demonstrated that MIR133a induces the downregulation of COL2A1 by targeting matrix metallopeptidase 9 (MMP9), causing the onset of IDD [34]; MIR146a suppresses IL-1β-induced MMP13, ADAM metallopeptidase with thrombospondin type 1 motif 4 (ADAMTS4), and ADAMTS5 expression in NP cells [35]; MIR98 or MIR27b downregulation contributes to the loss of COL2A1 in IDD [36,37]; MIR15a and MIR143 promote the progression of NP apoptosis by directly targeting BCL2 apoptosis regulator [38,39]; MIR15b silencing protects NP cells from IL-1β-induced ECM degradation by targeting SMAD3 [40]; MIR132 promotes ECM degradation [41] in human NP cells by direct targeting of growth differentiation factor 5 (GDF5, a member of TGFβ superfamily) which is also a target gene of MIR7 [37]; MIR210 promotes Fas-mediated apoptosis in human IDD by regulating the expression of homeobox A9 (HOXA9) [42]; MIR494 promotes ECM degradation and apoptosis of degenerative human NP cells by directly targeting SOX9 [43]; MIR10b is overexpressed in IDD and increased NP cell proliferation by targeting HOXD10 to derepress the RhoC-Akt signaling [44]; concerning miR-221, a recent paper investigated its role in the osteogenic differentiation of degenerated annulus fibrosus cells [45].

However, in this context it is important to consider at least the two issues described below. In vitro condition does not truly represent clinical scenarios since cell culture tends to cause the loss of the original cellular properties or select a particular cell type, therefore cell expansion required for large analysis does not often agree with defining the authentic tissue context response to a specific treatment. Therefore, the accumulation of certain characteristics and loss of others, that occur with increasing passage numbers, limit the clinical correlation. In vitro studies on isolated cellular sub-populations are useful for cell characterization, but poorly represent the ideal approach for understanding the response within an heterogeneous physiopathological microenvironment. Therefore, the results of many studies regarding the effectiveness of biological therapeutics as injectable anabolic factors able to slow or even reverse the degenerative trend of IDD need to be interpreted cautiously and carefully.

With this in mind, we tried to perform knockdown experiments on short - term culture of IVD primary cells. We established a relatively quick protocol, as described in the Material and Methods section, which allowed i. to produce P0 cells really resembling IVD microenvironment, ii. to minimize artifacts from extended in vitro culture, and iii. to obtain viable cells, by a mild enzymatic digestion to avoid completely disruption of ECM. We demonstrated that the silencing of miR-221 is able to drive the cells toward chondrogenic lineage increasing the expression levels of typical marker genes such as COL2A1, ACAN and SOX9.

Notably, antagomiR-221 treated cells restored higher levels of FOXO3 and TRPS1 attenuating the grade of severity based on a molecular point of view. The appearance of FOXO3 (a miR-221 validated target in other contexts) after antagomiR-221 treatment together with the demonstration that miR-221 directly targets FOXO3 also in IVD cells is an important evidence, since the expression of this crucial transcription factor significantly decreased in human degenerated discs [16,17].

Specific actions of FOXO proteins, in particular FOXO3, have been described in different contexts, including the protection against oxidative stress, the increase of expression of genes encoding proteins involved in DNA repair, the regulation of adult stem cell homeostasis, the inhibition of inflammatory cytokines production, and the extension of longevity [46]. For these reasons, interventions that increase FOXO3 level or activity is attracting considerable interest, and our evidence suggests that disc homeostasis may be preserved thanks to reactivation of FOXO3 by targeting miR-221.

Regarding TRPS1, which is involved in chondrogenesis and cartilage biology [15], we hypothesized that increase in its expression after miR-221 depletion may contribute to avoid undesired ossification in IVD microenvironment slowing down the degeneration process. In fact, calcification of cartilage cells is one of many events associated with IDD multifactorial disease, together with apoptosis, senescence, inflammation, and alterations in the ECM [2,47]. Therefore, since TRPS1 acts as a repressor of the function of RUNX2 [15,48], the master regulator of osteogenesis [20], the increased TRPS1 expression may contribute to maintain RUNX2 basal level and repress the RUNX2-mediated transactivation of genes associated with cartilage hypertrophy and ECM degradation, such as collagen type X alpha 1 chain (COL10A1), alkaline phosphatase (ALP), and MMPs [49], also in the IVD tissue.

Finally, we should also consider some limitations of this study as well as further investigation to better determine the role of miR-221 in human IDD. First, we did not define in detail the cause/effect relationship between the antagomiR-221 treatment and a large series of genes whose expression could be modulated. Here we focused on typical proteins such as COL2A1, ACAN and SOX9, whose loss is associated with intervertebral disc degeneration, and promising candidates such as FOXO3 and TRPS1 as potential positive modulators of IVD integrity, in order to demonstrate the successful phenotypic change following an effective antagomiR-221 treatment. However, what specific pathways are affected by miR-221 in IVD remain to be investigated through a global analysis of mRNA profile by RNA sequencing. Second, in order to shed light on certain aspects of disk disease and the potential for a biological therapy with antagomiR-221, further experiments with a larger sample size are needed to achieve a more reliable outcome. Moreover, we are planning to use highly appealing methods such as ex vivo organ culture systems with live disc cells, as recently proposed [50].

In conclusion, the present study demonstrates that a low number of cells obtainable from short - term culture of IVD primary cells, including mature and progenitor cells, allows to perform sufficient informative analysis, including silencing experiments and comparisons with P0 cell status. For the first time we have demonstrated that miR-221 may have a significant role in the etiology of IDD, suggesting that its down-regulation may play a pivotal role in preserving the disc homeostasis and in supporting the endogenous repair process. The scenario of biological treatment approaches for degenerated disc repair is widely expanding, and the use of specific molecules combined with adequate delivery systems into a degenerating disc appears promising [51]. Accordingly, the potential use of antagomiR-221 in clinical practice is an important challenge, and future experiments on animal models aimed at demonstrating the in vivo therapeutic effect, will serve to propose the use of this molecule as intradiscal injectable molecule.

## MATERIALS AND METHODS

### Patients and tissue samples

Surgical herniated human disc tissues were obtained from 16 donors (patients’ages were between 34 and 70 years, mean age 50 years, 11 male and 5 female) by using research protocol approved by Ethics Committee of the University of Ferrara and S. Anna Hospital (protocol approved on November 17th, 2016). During surgery through an anterior approach, the cervical disc was completely removed; sampling was obtained from the central core of the disc, in order to avoid anterior and posterior longitudinal ligament, as well as calcified portions of the discs. The level of disc degeneration was classified into two groups, namely mild and severe. Discs with mild degeneration are non homogeneous at Magnetic Resonance Imaging (MRI) with a hypointense dark gray signal intensity, present a dehydrated nucleus pulposus, soft consistency at surgery. Discs with severe degeneration present hypointense black signal intensity at MRI, a very dehydrated nucleus pulposus with calcifications, and hard consistency at surgery.

### Isolation of human IVD cells

Cervical intervertebral disc tissues (1-2 cm^3^) were collected, cut into small pieces, and subjected to mild digestion in 15 ml centrifuge tube with only 1 mg/mL type IV collagenase (Sigma Aldrich, St. Louis, USA) for 5 h at 37°C in Dulbecco’s Modified Eagle’s Medium (DMEM)/F12 (Euroclone S.p.A., Milan, Italy). The tube was shaken every 2 min and once the digestion was terminated, cell suspension was filtered with a Falcon™ 70 μm Nylon Cell strainer (BD Biosciences, Franklin Lakes, NJ, USA). Subsequently 300 xg centrifugation was conducted for 10 min, the supernatant discarded, the cells resuspended in basal medium (DMEM/F12 containing 15% fetal calf serum, 100 mg/mL streptomycin, 100 U/mL penicillin, and 1% Glutamine) (Euroclone) and seeded in polystyrene culture plates (Sarstedt, Nümbrecht, Germany) at 5000 cells/cm^2^. The cells that were released from the dissected tissue and maintained in culture at 37 °C in a humidified atmosphere with 5% CO_2_ within the first 48 hours were referred to as passage zero (P0) cells. P0 cells were expanded for a maximum of two passages by growing for a period not exceeding a week until subconfluent, detaching by trypsinization, and maintained in culture for two passages to obtain P2 cells. Where indicated the cells were expanded up to passage 4 (P4). At each passage the cells were subjected to cell morphology and gene expression analysis.

Freshly isolated chondrocytes from human nasal septum cartilage were obtained as previously described [10] and used as control cells.

### Flow cytometry

IVD progenitors were analyzed for the expression of mesenchymal and hematopoietic surface marker molecules, by direct immunofluorescent staining, as previously reported [10]. Briefly, cell pellets were resuspended in phosphate buffered solution (PBS), and incubated with fluorescein isothiocyanate (FITC)– or phycoerythrin (PE)–conjugated mouse anti-human antibodies CD14-FITC (#F0844; DakoCytomation; Dako, Glostrup, Denmark), CD44-PE, and CD105-PE (#550989, #560839; Becton Dickinson, Franklin Lakes, NJ, USA) for 15 min at 4 °C. Monoclonal antibodies with no specificity were used as negative control. Antibody-treated cells were then washed with PBS and spun down (300 xg). Cell pellets were resuspended in 400 μL of PBS and analyzed by FACS Scan (Becton Dickinson). For each sample, 20000 events were acquired and analyzed using the CellQuest software (Becton Dickinson).

### Cell transfection

P2 cells were seeded in polystyrene culture plates (1.82 cm^2^ area) (Sarstedt) until reaching 70% of confluence. After 24 h cells were transiently transfected with 30 nM antagomiR-221 (GAAACCCAGCAGACAAUGUAGCU) (Ambion Life Technologies, Grand Island, NY), a non-relevant antagomiR (antagomiR-Scr) (Ambion Life Technologies) in basal medium added with 20% Opti-MEM™ (ThermoFisher Scientific, Waltham, USA). For all transfections, Lipofectamine RNAiMAX (ThermoFisher) was used as delivering agent (0.43 μL/mL of culture medium) by combination with the oligonucleotides for 20 min at room temperature. The transfected cells were cultured for 48 h at 37 °C, in a humidified atmosphere at 5% CO_2_, then detached and used for in vitro experiments [11].

### Cell viability

Viability of the cells was assessed by Calcein-AM/propidium iodide (PI) staining (Cellstain double staining kit, Sigma-Aldrich). Before staining, the medium was removed from the wells, and 500 μL of the staining solution was added to each well. The samples were incubated in the dark at room temperature for 15 min, thereafter the wells were rinsed with PBS and immediately visualized under a fluorescence microscope (Nikon, Optiphot-2; Nikon Corporation, Tokyo, Japan). Dead cells stained red, while viable ones appeared green.

### Luciferase reporter gene assay

DNA fragment of the FOXO3 (507 bp) 3’-UTR to encompass miR-221 predicted target site, was inserted into the XhoI-XbaI restriction sites in the multiple cloning site of the reporter vector pmiRNano-GLO (Promega). This bicistronic vector contains NanoLuc luciferase (NlucP) as the primary reporter gene and Firefly luciferase (Luc2) as control reporter for normalization. Primers used in the PCR were, Forward FOXO3 3’-UTR: CCGCTCGAGTCCCTGCTTGAGTTCTTGCTGAT, which contains the XhoI restriction site (CTCGAG), Reverse FOXO3 3’-UTR GCTCTAGATTCACTGCTACTGGAAAGT, which contains the XbaI restriction site (TCTAGA). IVD cells were transfected with 100 ng of reporter vector in combination with 30 nM of pre-miR-221 precursor (named miR-221 mimic), or Negative control (all purchased from Ambion Life Technologies), using Lipofectamine 2000 reagent (ThermoFisher). After 48 hours, transfected IVD cells underwent NanoLuc and Firefly luciferase activity measurements using the GloMax 20/20 single tube Luminometer (Promega) and the Nano-Glo Dual-Luciferase Assay (Promega) according to the manufacturer’s recommendations. The ratio NanoLuc reporter activity/Firefly control reporter activity was calculated for each well. For each IVD sample all transfections were performed in triplicate, and data were presented as mean values with standard deviation.

### Immunocytochemistry

Immunocytochemistry analysis was performed employing the ImmPRESS (#MP-7500; Vectorlabs, Burlingame, CA). Cells grown in polystyrene culture plates were fixed in cold 100% methanol and permeabilized with 0.2% (v/v) Triton X-100 (Sigma-Aldrich) in TBS (Tris-buffered saline). Cells were treated with 3% H_2_O_2_ in TBS, and incubated in 2% normal horse serum (Vectorlabs) for 15 min at room temperature. After the incubation in blocking serum, the different primary antibodies were added and incubated at 4 °C overnight: polyclonal antibodies for COL2A1 (#Ab3092; mouse anti-human, 1:200 dilution, Abcam, Cambridge, UK), SOX9 (#sc-20095; rabbit anti-human, Santa Cruz Biotechnology), ACAN (#sc-33695; mouse anti-human, 1:200 dilution, Santa Cruz Biotechnology), TRPS1 (#20003-1-AP; rabbit anti-human, 1:100 dilution; Abcam), FOXO3 (#Ab70315; rabbit anti-human, 1:1000 dilution; Abcam), RUNX-2 (#sc-10758; rabbit anti-human, 1:100 dilution; Santa Cruz Biotechnology) and SOD2 (#sc-133134; mouse anti-human; 1:200 dilution; Santa Cruz Biotechnology). Cells were then incubated in Vecstain ABC (#MP-7500; Vectorlabs) with DAB solution (#SK-4105; Vectorlabs). After washing, cells were mounted in glycerol/TBS 9:1 and observed using a Leitz microscope (Wetzlar, Germany). Quantitative image analysis of immunostained cells was obtained by a computerized video-camera – based image-analysis system (with NIH, USA ImageJ software, public domain available at: http://rsb.info.nih.gov/nih-image/) under brightfield microscopy. Briefly, images were grabbed with single stain, without carrying out nuclear counterstaining with hematoxylin and unaltered TIFF images were digitized and converted to black and white picture to evaluate the distribution of relative gray values (i.e. number of pixels in the image as a function of gray value 0-256), which reflected chromogen stain intensity. Images were then segmented using a consistent arbitrary threshold 50% to avoid a floor or ceiling effect, and binarized (black versus white); total black pixels per field were counted and average values were calculated for each sample. Three replicate samples and at least ten fields per replicate were subjected to densitometric analysis. We performed the quantification of pixels per 100 cells and not per area in order to take into account the different cell morphology and confluence.

### RNA extraction and quantitative real-time (qRT)-PCR

Total RNA, including miRNAs, was extracted from IVD cells using the RNeasy Micro Kit (Qiagen, Hilden, Germany), according to the manufacturer’s instructions. RNA concentration and quality was measured using a NanoDrop ND1000 UV-VIS spectrophotometer (Isogen Life Science, de Meern, the Netherlands). cDNA was synthesized from total RNA in a 20 μL reaction volume using the TaqMan MicroRNA Reverse Transcription kit (ThermoFisher) for analysis of microRNAs, or the TaqMan High Capacity cDNA Reverse Transcription kit (ThermoFisher) for analysis of mRNAs. Quantification of miR-221 was performed using the TaqMan MicroRNA Assays (ThermoFisher), using U6 snRNA for normalization. For the quantification of COL2A1, ACAN, SOX9, FOXO3 and TRPS1 mRNA, the appropriate TaqMan Assays were purchased (ThermoFisher); Glyceraldehyde 3-phosphate dehydrogenase (GAPDH) gene was used for normalization of mRNA abundance. Polymerase chain reactions were performed with the TaqMan Universal PCR MasterMix (ThermoFisher) using the CFX96TM PCR detection system (Bio-Rad, Hercules, CA, USA). Relative gene expression was calculated using the comparative 2-ΔΔCt method (expressed as fold change). All reactions were performed in triplicate and the experiments were repeated at least six times.

### Western blot analysis

Total cell extracts were prepared from IVD cells as previously reported [10]. 20 μg of each sample were electrophoresed through a 4-15% SDS-polyacrylamide gradient gel. The proteins were then transferred onto an Immobilon-P PVDF membrane (Millipore, Billerica, MA). After blocking with TBS-0.1% Tween-20 and 5% nonfat dried milk (Sigma-Aldrich), the membrane was probed with monoclonal mouse anti-human FOXO3 antibody (#sc-48348, clone D-12; 1:1000 dilution) (Santa Cruz Biotechnology). After washing with TBS-0.1% Tween 20, the membranes were incubated with the appropriate horseradish peroxidase conjugated secondary antibody (Dako, Glostrup, Denmark). Immunocomplexes were detected using Immobilon Western Chemiluminescent HRP Substrate (Merck-Millipore). A mouse monoclonal anti-β-actin antibody (Sigma-Aldrich) was used for normalization. Densitometric analysis was performed by ImageJ software (NIH, USA, public domain available at: http://rsb.info.nih.gov/nih-image/).

### Histochemical analysis

Small fragments of each IVD sample were rinsed with PBS, fixed in 4% buffered paraformaldehyde for 24 h at 4 °C, embedded in paraffin and cross-sectioned (5-μm thick). For histological evaluation non consecutive sections were stained with 0.1% Safranin-O solution or immunostained with TRPS1 (#20003-1-AP; rabbit anti-human, 1:100 dilution; Abcam) and FOXO3 (#Ab70315; rabbit anti-human, 1:100 dilution; Abcam). Immunohistochemical sections were deparaffinized, rehydrated, and enzymatically treated with 20 μg/mL of proteinase K. Slides were then processed with 0.3% H_2_O_2_ in PBS 1X for 5 min and with blocking solution (PBS 1X/ 1% BSA/ 10% FCS) for 30 min at room temperature. Then the slides were incubated over night with the primary antibody at 4 °C, followed by treatment with Vecstain ABC reagent (Vectorlabs) for 30 min. The reactions were developed using DAB solution (Vectorlabs), the sections were counterstained with hematoxylin and mounted in glycerol. The stainings were quantified by a computerised video camera-based image analysis system (NIH, USA ImageJ software, public domain available at: http://rsb.info.nih.gov/nih-image/) under brightfield microscopy (NikonEclipse 50i; Nikon Corporation, Tokyo, Japan), as reported above. For the analysis of sections, positive cells in the area were counted and protein levels expressed as % of positive nuclei (ten fields per replicate, 5 sections per sample).

### Statistical analysis

Data are reported as mean value ± standard deviation (SD). Comparison between two groups were assessed by unpaired Student’s t test. All statistical analyses were performed using Prim 6 software (GraphPad Software). P-values less than 0.05 were considered significant.
